# Conjunctival macrophages act as antigen-presenting cells in the conjunctiva during the development of experimental allergic conjunctivitis

**Published:** 2010-07-10

**Authors:** Waka Ishida, Ken Fukuda, Mina Kajisako, Ayako Takahashi, Tamaki Sumi, Nico van Rooijen, Atsuki Fukushima

**Affiliations:** 1 Department of Ophthalmology, Kochi Medical School, Nankoku-city, Japan; 2 Department of Cell Biology and Immunology, Faculty of Medicine, Vrije Universiteit, Amsterdam, The Netherlands

## Abstract

**Purpose:**

Antigen (Ag)-presenting cells (APCs) participate in the development of allergic conjunctivitis (AC). However, the conjunctival APCs that take up Ag in AC have not been identified. We sought to clarify the phenotypes of conjunctival APCs that take up, process, and present Ag to T cells during the development of experimental AC.

**Methods:**

Splenocytes from naïve ovalbumin (OVA)-specific T cell receptor transgenic (DO11.10) mice were stimulated with either OVA, gold, or OVA-gold to evaluate stimulation-induced proliferation. Naïve DO11.10 mice were subconjunctivally injected with PBS, gold, or OVA-gold. Twenty-four hours later, conjunctivas were harvested for immunohistochemistry and electron microscopy to identify cells that engulfed OVA-gold.

**Results:**

Stimulation of DO11.10 splenocytes with OVA-gold but not gold alone induced similar levels of proliferation as OVA alone. Subconjunctival injection of OVA-gold, but not gold alone, induced infiltration of eosinophils and CD4-positive T cells into the conjunctiva. The cells that took up OVA-gold into their cytoplasma expressed Cluster of Differentiation (CD) 11b, CD68, major histocompatibility complex (MHC) class II.

**Conclusions:**

It appears that conjunctival macrophages are APCs in the development of experimental AC.

## Introduction

Allergic conjunctivitis (AC) is mediated by activation of mast cells and the released chemical mediators [1]. These mediators are capable of inducing infiltration of inflammatory cells into the conjunctiva. We demonstrated that antigen (Ag)-specific immunoglobulin (Ig) E -mediated mast cell activation in the conjunctiva is not able to induce severe conjunctival infiltration of eosinophils [2], a hallmark of severe forms of AC [3]. In contrast, transfer of Ag-specific T helper type 2 (Th2) cells into syngeneic mice followed by Ag challenge in the conjunctiva induced significant infiltration of eosinophils [4]. Thus, it appears that Th2 cells rather than mast cells play an important role in conjunctival eosinophilia during the development of experimental AC (EC).

For Th2 cell activation, Ag-presenting cells (APCs) present Ag peptides in the context of major histocompatibility complex (MHC) class II [5]. In AC, Ag challenge occurs in the conjunctiva, and the Ag must be captured by conjunctival APCs. We previously investigated the identity of conjunctival APCs during the development of EC by using Brown Norway rats [6]. In that report, we were not able to confirm which conjunctival APCs take up and process the Ag due to the limited availability of reagents in this system. Therefore, in this study, we sought to clarify the phenotypes of conjunctival APCs that take up and process Ag by using the Ag conjugated with gold as a marker.

## Methods

### Mice

αβ-T cell receptor (TcR)-transgenic BALB/c DO11.10 mice [7], which are transgenic for the TcR specific for the immunodominant epitope of ovalbumin (OVA; peptide 323–339), were kindly provided by Dr. Iwabuchi (Hokkaido University, Japan). They were bred in a specific pathogen-free animal facility in Kochi Medical School. Animals between 6 and 12 weeks of age were used for the experiments. All animal procedures conformed to the ARVO Resolution on Use of Animals in Research.

### Reagents

OVA (grade V) was purchased from Seikagaku Co.,Tokyo, Japan. Gold (diameter: 15 nm) was purchased from BB International (Cardiff, UK). Anti-mouse Cluster of Differentiation 11b (CD11b) (Invitrogen Corp, Carlsbad, CA), anti-mouse Cluster of Differentiation 4 (CD4), anti-mouse Cluster of Differentiation 68 (CD68), and anti-MHC class II (BD Biosciences PharMingen, San Diego, CA) were purchased, and rabbit anti-mouse major basic protein (MBP) was kindly provided by Dr. James J. Lee (Mayo Clinic, Scottsdale, AZ). Biotinylated rabbit polyclonal anti-rat immunoglobulins (Dako Cytomation, Glostrup, Denmark) and biotinylated goat polyclonal anti-rabbit immunoglobulins (BD Biosciences PharMingen) were used as secondary antibodies (Abs).

### Preparation of OVA-gold

To conjugate OVA to gold, 18 ml of gold colloid was reacted with 720 μg OVA. The suspension was then centrifuged, and the supernatant was discarded. The final concentration of OVA in the OVA-gold conjugate was calculated to be 1.67 mg/ml. The number of gold particles was 5×10^12^ per ml. These procedures were conducted in Operon Biotechnologies Japan, Tokyo, Japan.

### Cellular proliferation assay

3×10^5^ splenocytes/well from naïve DO11.10 mice were cultured with 1, 10, and 100 μg/ml of OVA in 96-well flat-bottom plates in a final volume of 0.2 ml RPMI 1640 medium supplemented with 5% fetal calf serum (FCS) and 2-mercaptoethanol (2-ME). As a background control, OVA, gold, and OVA-gold were omitted from the well. OVA-gold was added to the culture at OVA concentrations of 1, 10, and 100 μg/ml. Gold alone was added to the culture so that the wells contained the same amount of gold as the OVA-gold wells. After incubation for 48 h at 37 °C in a humidified atmosphere with 5% CO_2_, cultures were pulsed for 16 h with 0.5 μCi/well of [^3^H]thymidine (Japan Atomic Energy Research Institute, Tokai, Japan). The cultures were then harvested and the radioactivity was measured by standard techniques. Data were expressed as mean cpm+/−SD.

### Immunohistochemistry

DO11.10 mice were subconjunctivally injected wtih PBS, gold or OVA-gold (n=4 mice per group). The mice were injected with 10 μl of OVA-gold containing 16.7 μg of OVA and 5×10^9^ gold particles. When gold alone was administered, mice were injected with the same amount of gold that was present in the OVA-gold injections. Twenty-four hours later, the conjunctivas were harvested, embedded in Optimal Cutting Temperature (OCT) compound (VWR, Suwanee, GA) and snap frozen in liquid nitrogen. Vertical 4-μm sections were prepared and fixed in methanol. Endogenous peroxidase activity was inhibited by incubation with 0.1% NaN_3_ and 0.3% H_2_O_2_ in distilled water for 10 min at room temperature. The samples were exposed to the anti-MBP, anti-CD4, anti-CD11b, or anti-CD68 Abs for 30 min and then to the appropriate biotinylated secondary Ab for another 30 min. As a control, the samples were exposed to biotinylated rabbit polyclonal anti-rat immunoglobulins but not to any first Abs. Ab binding was revealed by using an Avidin-Biotin-Complex kit (Vector Laboratories Inc., Burlingame, CA) followed by development with 3,3′-diaminobenzidine tetrahydrochloride (Sigma, St. Louis, MO).

### Transmission electron microscopy

The conjunctivas were harvested and fixed in 2.5% glutaraldehyde, 2.0% paraformaldehyde, and 0.1 M phosphate buffer (pH 7.2), immersed in 1% OsO_4_ for 1 h at 4 °C, stained en bloc with 2.0% uranyl acetate for 30 min, dehydrated by graded concentrations of ethanol, and embedded in Spurr's resin. Thin sections (90 nm) were generated and stained with either methylene blue-azure II-basic fuchsin [8] or uranyl acetate and lead citrate to examine under light microscope or electron microscope (H7100; Hitachi, Tokyo, Japan), respectively.

### Statistical Analysis

Because the infiltrating cell number data and the proliferation assay data showed approximately normal distribution (data not shown), the parametric test was applied for statistical analysis. P values <0.05 were considered to be significant.

## Results

### OVA-gold stimulates splenocytes from DO11.10 mice

**Figure 1:**
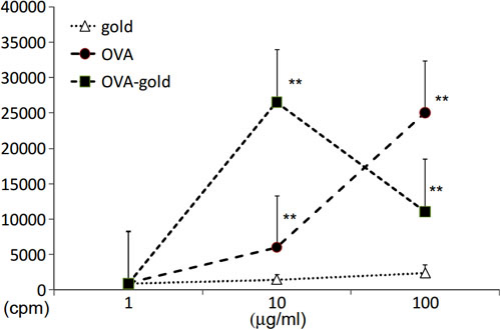
In vitro antigenicity of OVA-gold. Splenocytes from naïve DO11.10 mice were cultured in vitro with OVA, gold, or OVA-gold. OVA-gold was added to the culture at a concentration of 1, 10, and 100 μg/ml of OVA . Gold alone was added to the culture so that the same amount of gold per well was added as in the OVA-gold wells. Proliferation was evaluated by [^3^H]thymidine incorporation. Background cpm (no Ag) was 1707+/−290. One representative data of two experiments is shown. The double asterisk indicates a p<0.01 compared to the background.

To examine whether OVA-gold has antigenicity, splenocytes from naïve DO11.10 mice were stimulated in vitro with OVA-gold. As controls, these splenocytes were stimulated with either OVA alone or gold alone. Similar to when splenocytes were stimulated with OVA alone, OVA-gold induced significant proliferation of splenocytes both at 10 and 100 μg/ml (Figure 1). For OVA-gold stimulation, as the OVA concentration in vitro increases, amount of gold in each well also increases. To examine whether the amount of gold affects splenocyte proliferation, we added gold alone at a level identical to that in OVA-gold at three different OVA concentrations (1, 10, 100 μg/ml). Regardless of the amount of gold added to the well, gold alone did not significantly induce splenocyte proliferation (Figure 1).

### Subconjunctival injection of OVA-gold induces conjunctival inflammation

**Figure 2:**
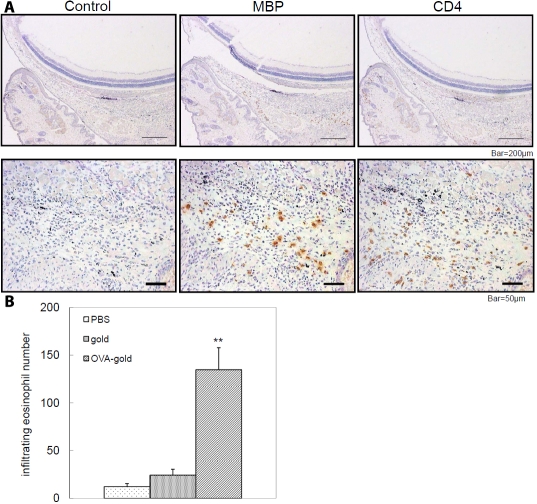
Effect of subconjunctival injection of OVA-gold in the conjunctiva. OVA-gold, gold or PBS (10 μl) was injected into the subconjunctival space of DO11.10 mice. Twenty-four hours later, conjunctivas were collected for immunohistochemical analyses. MBP-positive eosinophils and CD4-positive T cells were detected in the OVA-gold-injected group (**A**). Control samples were not stained with any primary antibodies. MBP-positive eosinophils were quantified in the OVA-gold and control groups (**B**). The double asterisk indicates a p<0.01 compared to PBS and the gold groups.

Next, we investigated whether OVA-gold has antigenicity in vivo. To this end, OVA-gold was injected into the subconjunctival space of DO11.10 mice. Subconjunctival injection of OVA-gold induced an influx of inflammatory cells in the conjunctiva and immunohistochemical analysis of conjunctival infiltrating cells revealed that MBP-positive eosinophils and CD4-positive T cells infiltrated into the conjunctiva of OVA-gold-injected mice (Figure 2A). Subconjunctival injection of gold alone or PBS did not induce apparent inflammation in the conjunctiva (data not shown). Counting of MBP-positive eosinophils revealed that significantly more eosinophils were detected in the conjunctiva of the OVA-gold-injected group compared to the gold alone- or PBS-injected groups (Figure 2B). These results indicate that OVA-gold has antigenicity in vivo as well as in vitro.

### Subconjunctivally injected OVA-gold exists in the cytoplasma of cells in the conjuncitva

**Figure 3:**
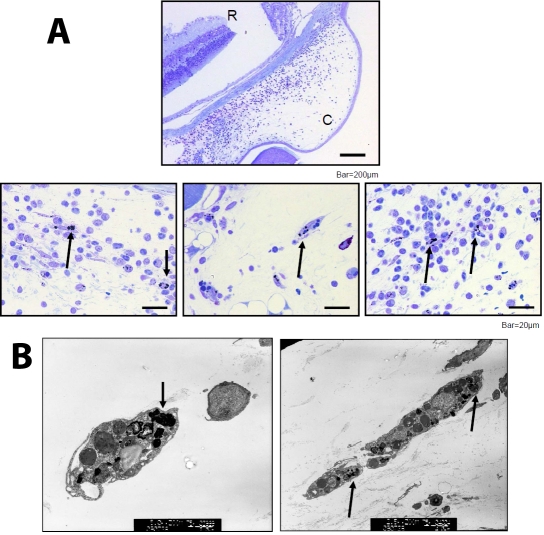
Examination of cells that engulfed OVA-gold in the conjunctiva. Conjunctival samples were prepared as described in Figure 2. Cells that engulfed OVA-gold were examined histologically by light (**A**) and electron microscopy (**B**). Gold particles were detected in the cytoplasma of cells in the conjunctiva (**A**). C and R indicates conjunctiva and retina, respectively (**A**). Gold particles were present in cytoplasms of oval-shaped cells and spindle-like-shaped cells (**B**). Arrows indicate cells that engulfed gold particles.

The above findings suggest that OVA-gold was engulfed by cells in the conjunctiva, and antigenic epitopes were presented to OVA-specific T cells. To examine this directly, conjunctivas were harvested 24 h after subconjunctival injection of OVA-gold. These conjunctivas were subjected to histological analysis by using either a light microscope or an electron microscope. Light microscopic analysis demonstrated that gold particles were detected in the cytoplasm of cells in the conjunctiva (Figure 3A). Gold particles were also detected in the extracelluar matrix spaces (data not shown). Electron microscopic analysis revealed that gold particles were present and had accumulated in the cytoplasm of oval-shaped cells and spindle-like-shaped cells (Figure 3B).

### Cells engulfed OVA-gold express macrophage surface markers

**Figure 4:**
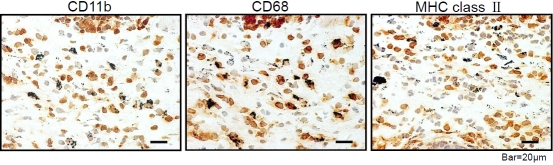
Phenotypic characterization of cells that engulfed OVA-gold in the conjunctiva. Conjunctival samples were prepared as described in Figure 2. Samples were evaluated immunohistochemically to examine the expression of CD11b, CD68, and MHC class II.

To further characterize the phenotypes of the cells that took up OVA-gold, harvested conjunctivas were subjected to immunohistochemical analysis. Cells that engulfed OVA-gold express CD11b, CD68, and MHC class II molecules (Figure 4).

## Discussion

Although there have been several reports [9-13] that investigated APCs in the conjunctiva of AC patients and animals, none confirmed which cell types take up the Ag and present antigenic peptides to T cells. Using gold as a marker, we sought to identify the conjunctival APCs that play a crucial role in the development of AC.

We found that after subconjunctival injection of OVA-gold, CD11b- and CD68-positive cells in the conjunctiva took up the conjugated particles. Importantly, MHC class II was expressed in the cells that engulfed OVA-gold. Thus, MHC class II-expressing macrophages are likely to be the APCs in the conjunctiva.

The uptake of OVA-gold by conjunctival macrophages does not guarantee that the macrophages that took it up express antigenic peptides to T cells. To examine this issue, we initially investigated the antigenicity of OVA-gold using DO11.10 splenocytes which contain OVA 323–339-specific T cells. OVA-gold, as well as OVA, induced proliferation of splenocytes in a dose-dependent manner, while gold alone did not. Thus, it was confirmed that OVA-gold has similar antigenicity to OVA. Next, we investigated antigenicity in vivo by injecting OVA-gold subconjunctivally into DO11.10 mice. OVA-gold, but not gold alone, induced conjunctival eosinophilia to a similar severity as resulted from subconjunctival injection with OVA alone. Thus, it appears that OVA-gold is antigenic both in vitro and in vivo. Together with the fact that the OVA-gold-engulfing, CD68-positive macrophages also express MHC class II molecules, these data show that this cell population indeed functions as an APC in the conjunctiva during the development of EC.

To investigate the role of conjunctival macrophages in conjunctival eosinophilia, clodronate liposomes [14] were injected into the conjunctiva of DO11.10 mice. Seventy-two hours later when conjunctival macrophages were depleted, OVA-gold was subconjunctivally injected and 24 h later, conjunctivas were collected for immunohistochemical analysis to detect eosinophils. Conjunctival eosinophil numbers were not different between mice that received clodronate liposomes and PBS as a control (data not shown). This result suggests that not only macrophages but also other types of cells such as dendritic cells are APC in the conjunctiva. Another possibility is depletion of conjunctival macrophages is not complete as previously reported [15] and therefore, residual macrophages uptake OVA-gold and present the Ag to T cells.

It is possible that a certain type of APC may not be able to uptake a large particle such as gold. It is also possible that some types of APCs can take up OVA, but not OVA-gold. Although CD68-positive macrophages were identified as APCs in the conjunctiva during the development of EC in this study, other types of APC may also play a role, as discussed above. In the future, the use of small molecules rather than gold will aid in clarifying the role of dendritic cells in the development of EC. Furthermore, our data support the idea that APC-targeted therapy may be useful for the prevention of AC, since dysfunction of conjunctival APCs attenuates T cell activation in the conjunctiva.
